# Differential Elevation of Inflammation and CD4^+^ T Cell Activation in Kenyan Female Sex Workers and Non-Sex Workers Using Depot-Medroxyprogesterone Acetate

**DOI:** 10.3389/fimmu.2020.598307

**Published:** 2021-02-23

**Authors:** Kenneth Omollo, Julie Lajoie, Julius Oyugi, Jocelyn M. Wessels, Dufton Mwaengo, Joshua Kimani, Charu Kaushic, Keith R. Fowke

**Affiliations:** ^1^ Department Medical Microbiology, University of Nairobi, Nairobi, Kenya; ^2^ Partners for Health and Development in Africa, Nairobi, Kenya; ^3^ Department of Medical Microbiology and Infectious Diseases, University of Manitoba, Winnipeg, MB, Canada; ^4^ McMaster Immunology Research Centre, Michael G. DeGroote Centre for Learning and Discovery, McMaster University, Hamilton, ON, Canada; ^5^ Department of Pathology and Molecular Medicine, McMaster University, Hamilton, ON, Canada; ^6^ Department of Community Health Science, University of Manitoba, Winnipeg, MB, Canada

**Keywords:** DMPA, inflammation, T-cell activation, HIV target cells, endocervix, commercial sex workers

## Abstract

**Background:**

Depot Medroxyprogesterone (DMPA) is one of the most widely used contraceptives in Sub-Saharan Africa where HIV incidence is high. We explored the effect of DMPA on the activation of HIV cellular targets and inflammation as a possible mechanism of increased HIV risk with DMPA use. Since sex work is known to affect the immune system, this study aimed to understand the effect of DMPA on the immune system among sex workers and non-sex worker women.

**Methods:**

Twenty-seven DMPA-using HIV seronegative female sex workers (FSW) and 30 DMPA-using HIV seronegative non-sex worker (SW) women were enrolled in the study. Twenty-four FSWs and 30 non-sex workers who were not using any hormonal contraception (no HC) were recruited as controls. Blood and cervico-vaginal samples were collected from all participants and assayed for T cell activation and proinflammatory cytokines.

**Results:**

Among no HC users, sex workers had lower expression of CD38 and CD69 on blood-derived CD4^+^ T cells along with lower CD4^+^CCR5^+^ cells frequency in the endocervix. Plasma MCP-1, TNFα and IL-17 also had reduced expression in FSW not using HC. Non-sex workers using DMPA had elevated proportions of blood-derived CD4^+^CD38^+^, CD4^+^CD69^+^ and CD4^+^HLA-DR^+^ T cells relative to non-sex workers who were not taking any HC. DMPA-using non-sex workers also had an increased level of plasma interferon gamma (IFN-*γ*), monokine induced by interferon-*γ* (MIG) and sCD40L, alongside higher proportion of CD4^+^CD38^+^ and CD4^+^CD69^+^ T cells at the cervix compared to non-sex workers no-HC controls., Finally, non-sex workers and FSWs using DMPA had similar levels of genital and peripheral CD4^+^ T cell activation and inflammation.

**Conclusion:**

DMPA increased inflammation and expression of activation markers on potential HIV target cells in non-sex workers. These data show that DMPA is a strong immune modulator and its use counteracts the decreased immune activation associated with sex work. These findings suggest that inflammation and increased HIV target cells in blood and at the genital tract may be mechanisms by which DMPA increases susceptibility to HIV.

## Introduction

Safe and effective contraception is a critical component of basic healthcare for women. Unfortunately, a number of observational studies have associated depot medroxyprogesterone acetate (DMPA), with a higher risk of HIV-1 acquisition (1–5). DMPA is a low-cost, progestin-based contraceptive administered as a 3-monthly intramuscular (IM) injection and is the most widely used reversible contraceptive among young women living in sub-Saharan Africa (SSA) (6). Importantly, this region also bears the highest burden of HIV worldwide (7). Therefore, an association between DMPA use and HIV acquisition in this region is of public health concern.

In an attempt to clarify this association, the Evidence for Contraceptive Options and HIV Outcomes (ECHO) study was conducted in four SSA countries. ECHO was an open-label trial comparing HIV incidence among women randomized to DMPA-IM, copper Intrauterine Device (IUD), or levonorgestrel (LNG) implant (8). The trial found no statistically significant differences in HIV incidence among women in the 3 study arms. However, HIV incidence was “alarmingly high” in all 3 groups (8) and the study did not include a no hormonal contraception (HC) control group. Therefore, the issue of increased HIV risk due to HC use remains unresolved.

The possible effect of DMPA on HIV acquisition may result from changes in sexual behavior and/or biological factors. One of the potential biological mechanisms is that DMPA may increase the number of HIV target cells, especially CD4^+^CCR5^+^ T cells, at the female genital tract (FGT), which is the major route of infection in women (9, 10). It has been observed that DMPA also increased the numbers of CD3^+^ expressing HLA-DR and CCR5 cells in vaginal tissues, leading to more activated target cells for the virus to infect (11). Furthermore, a recent study from our group demonstrated that sex workers using DMPA had higher levels of resident activated (CD4^+^CD69^+^) T cells and HIV target cells (CD4^+^CCR5^+^) in the genital tract than those not using any HC (12). Taken together, these findings suggest that increased expression of CCR5 and T cell activation may contribute to an epidemiological link between DMPA and HIV acquisition.

Increased concentrations of inflammatory cytokines and reduced secretion of innate antiviral proteins at the FGT are associated with heightened risk of HIV infection. *Ex vivo* treatment of endometrial biopsies with DMPA results in decreased expression of the antimicrobial protein Secretory Leukocyte Protease Inhibitor (SLPI) (13). Decreased SLPI levels have been associated with increased risk of HIV seroconversion in women (14). Cervical cells treated with MPA show an increased gene expression of Interleukin(IL)-8 and decrease in CCL5 – a competitive inhibitor of CCR5 on cells (15). By modifying the inflammatory and/or chemotactic milieu of the FGT mucosa, DMPA may increase the recruitment of HIV-target cells to the mucosa and therefore the risk of HIV acquisition (16, 17). However, concentrations of DMPA used in these *in vitro* studies exceeded physiological levels, which highlights the need for clinical studies.

Defining the impact of DMPA on the FGT immune response is important in understanding the biological factors influencing the risk of HIV acquisition in women. Furthermore, while others (18) and ourselves (19, 20) have demonstrated the strong immune-modulating impact that sex work has on the immune response, it remains unknown whether the impact of DMPA would be different between FSW and women from the general population. Understanding if DMPA differentially impacts the immune response of a high-risk population compared to a general population is important to help women at higher risk of HIV acquisition access better counseling on how to prevent pregnancy and protect themselves against HIV. Therefore, the aim of this study was to compare levels of T cell activation and concentrations of proinflammatory cytokines/chemokines in blood and the genital mucosa of women (FSW and non-sex workers) using DMPA and those not using any hormonal contraceptive.

## Materials and Methods

### Study Population

FSWs were recruited from the Pumwani Sex Worker Cohort at the Majengo Clinic in Nairobi, Kenya. Women from the general population (non sex workers: controls) living in the same community were recruited at the nearby Babadogo and Pumwani Health Centers. Recruitment was carried out between November 2015 and April 2018. FSWs were included in the study if they had been practising sex work less than 5 years by self-declaration. The Kenyatta National Hospital-University of Nairobi Ethical Review Committee (Nairobi, Kenya) and the Institutional Review Board at the University of Manitoba (Winnipeg, Canada) and McMaster University (Hamilton, Canada) approved the study. Written informed consent was obtained from all participants.

### Study Procedures

This study is a sub-study of a parent study looking into the impact of DMPA on the vaginal microbiome (21). Enrolment strategy as well as inclusion and exclusion criteria have already been reported (21). Briefly, prior to enrolment, consenting participants were screened for eligibility. Participants were considered for enrolment if they were: adults >18 years old, testing HIV-negative, pre-menopausal, not pregnant or breastfeeding, willing to undergo pelvic exams, had an intact uterus and cervix, were in general good health, testing negative for classical STIs (gonorrhea, chlamydia, trichomonas and syphilis), did not have a yeast infection and had a Nugent Score <7. To control for the possible immunomodulatory effect of seminal fluid on the FGT, participants were advised to use condoms for any sexual activities 36 h following the pre-screening visit and afterwards to abstain from sexual activities 12 h prior to the enrolment visit where samples were collected. Sexual abstinence was confirmed using an immunochromatographic test for Prostate Specific Antigen (Seratec PSA Semiquant, Göttingen, Germany). Participants who tested positive for PSA were excluded from the study.

Women using DMPA were included if they had been on DMPA for at least 6 consecutive months. Women not using hormonal contraception were recruited in the follicular phase of the menstrual cycle and should not have used any hormonal contraceptive in the preceding 6 months. At the enrolment visit, a clinical, demographic and behavioral questionnaire was completed by all participants.

### Sample Collection

Blood was collected through venipuncture with sodium heparin as an anticoagulant. Peripheral blood mononuclear cells (PBMCs) and plasma were freshly isolated using the Ficoll-Hypaque technique. Cervical mononuclear cells (CMCs) were collected under speculum examination using a cytobrush and processed as previously described (22). Briefly, the tubes containing cytobrush and scraper were vortexed for 1 min to dislodge cells from the brushes. Two washes were performed with 5ml RPMI and 5ml PBS consecutively, each wash followed by 10-min centrifugation. Staining of the CMC pellet for flow cytometry then proceeded.

Cervicovaginal lavages (CVL) were collected by washing the endocervical os with 2 ml of sterile 1x phosphate-buffered saline (PBS) and fluid was collected from the posterior fornix into a 15ml conical tube before being centrifuged to remove cellular debris. CVL supernatants and plasma were stored at −70°C prior to shipping to Winnipeg, Manitoba.

### PBMC and CMC Phenotyping

For each participant, 10^6^ PBMCs and the whole CMC pellet were stained for *ex vivo* immunophenotyping. First, fresh cells were stained for 30 min at 4°C with ECD-Live-Dead discriminant dye (Invitrogen, Carlsbad, USA) to identify viable cells. The cells were washed twice with FACS Buffer (2% FBS-1x PBS) then suspended in blocking solution (mouse IgG, FACS Buffer, FBS) for 10 min at 4°C followed by another wash. A mastermix of PECy5-CD3, Alexa700-CD4, BV510-HLA-DR, PECy7 -CD69, BV421-CCR5, and PE-CD38 and Brilliant Violet Stain Buffer (BD Biosciences, San Jose, USA) was used in labeling the cells for 30 min at 4°C. Cells were then washed and fixed in 1% paraformaldehyde. Data were acquired on an LSRII flow cytometer (BD System, San Jose, USA) and analyzed using FlowJo v10.0.8r1 (TreeStar, Ashland, USA). To control for the quality of the data, CMC samples having <100 viable CD3^+^ cells were excluded from the analyses. This is a standard quality control method used in our laboratory (12).

### Cytokine and Chemokine Measurement

Cytokine and chemokine concentration in plasma and CVL were determined using the Milliplex MAP kit (Millipore, Billerica, MA) and analyzed on the BioPlex-200 (Bio-Rad, Mississauga, ON, Canada). CVL samples were assayed using the overnight incubation protocol, while plasma samples were assayed using the 2-h incubation protocol according to the manufacturer and as previously as described by Lajoie et al (23). Proteins investigated were: IL-1α, IL-1Rα, IL-1β, IL-8, IL-10, IL-17, Interferon (IFN)-γ, Tumor Necrosis Factor (TNF), Monocyte Chemoattractant Protein (MCP)-1, Macrophage Inflammatory Protein (MIP)-1, MIP-1β, IP-10, Monokine Induced by gamma (MIG) and MIP-3a.

### Statistical Analysis

Analyses were performed using GraphPad Prism 7.0 (GraphPad Software, La Jolla, CA). Gaussian distribution was tested by the D’agostino and Pearson’s omnibus normality test and Shapiro–Wilk normality test. The Mann – Whitney *U-* test was used to compare variables that were not normally distributed between the two groups. The Chi-square and Fisher’s Exact tests were used to assess the significance of the associations between categorical demographic variables. Statistical significance was accepted if *p ≤ 0.05.*

## Results

### Demographics

**Figure 1:**
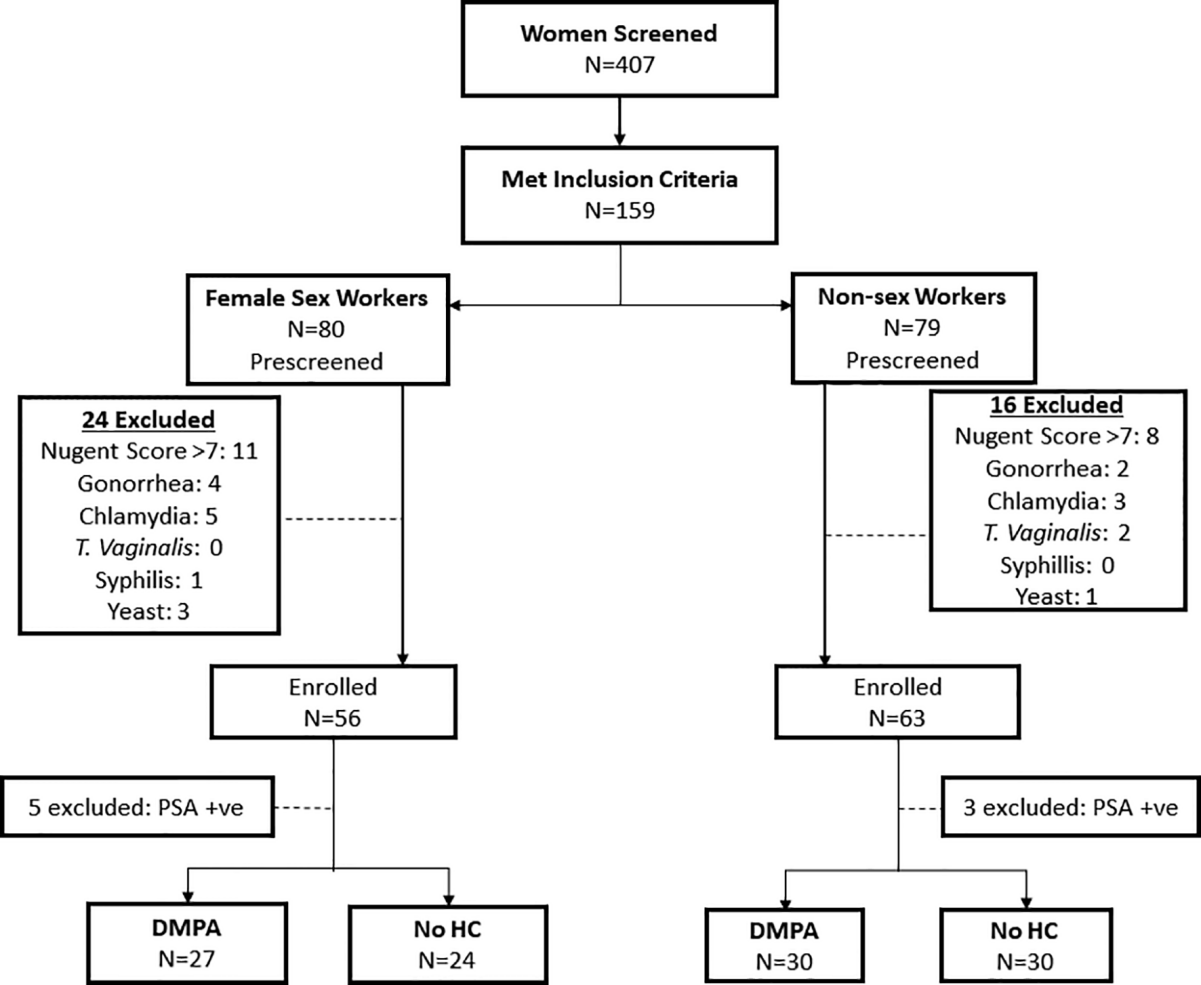
Study Profile. Flow chart depicting the screening and enrolment process for study participants.

Fifty-one (51) FSWs (27 on DMPA and 24 not using HC) and 60 non-sex workers (30 on DMPA and 30 not using any HC) who met the criteria were included in the study (**Figure 1**). **Table 1** summarizes the clinical and demographic characteristics of the study participants. No differences were observed within the two groups of FSWs and non-sex workers in age. Non-sex workers using no HC were likely to have more years of education than their counterparts using DMPA (p<0.001) with the majority having high school and college level education. In non-sex worker groups, DMPA use was predominantly (>75%) in women who were in married relationships. Less than 25% of non-sex workers in non-committed relationships reported use of DMPA. Due to the enrolment criteria, the groups were similar in Bacterial Vaginosis (BV) profile. Both groups were similar for alcohol use.

**Table 1:** Sociodemographic and clinical characteristics of participants.

	Female Sex Workers			Non-Sex Workers		
	DMPA	No HC	*p-value*	DMPA	No HC	*p-value*
	N=27	N=24		N=30	N=30	
**Age: Years, Mean (Range)^a^**	27(18–39)	30(19–39)	0.082	29(20–43)	28(18–46)	0.185
**Years of school: Mean**	10(3–17)	10(6–14)	0.395	9(6–14)	12(6–15)	**<0.001**
**Education level^b^**						
Primary	10(37.1%)	9(37.5%)	0.990	20(66.7%)	3(10%)	**<0.001**
Secondary	15(55.5%)	13(54.2%)		9(30.0%)	15(50.0%)	
College	2(7.4%)	2(8.3%)		1(3.3%)	12(40.0%)	
**Marital status^b^**						
Not married, not living with a man	17(63.0%)	16(66.7%)	0.413	1(3.3%)	23(76.6%)	**<0.001**
Not married, living with a man	2(7.4%)	4(16.6%)		–	–	
Married, not living with a man	7(25.9%)	4(16.7%)		6(20%)	2(6.7%)	
Married, living with a man	1(3.7%)	0(0.0%)		21(70.0%)	5(16.7%)	
Widowed, not living with a man	–	–		2(6.7%)	0(0.0%)	
**BV diagnosis^b^**						
Normal (Nugent score 0–4)	17(63.0%)	18(75.0%)	0.646	24(80.0%)	23(76.7%)	0.754
Intermediate (Nugent score 5–7)	7(25.9%)	4(16.7%)		6(20.0%)	7(23.3%)	
Undetermined	3(11.1%)	2(8.3%)		–	–	
**Alcohol use^b^**						
No	13(48.2%)	13(54.2%)	0.781	26(86.7%)	21(70.0%)	0.209
Yes	14(51.8%)	11(45.8%)		4(13.3%)	9(30.0%)	
**Parity^c^**						
No. of children	2(0–4)	2(0–4)	0.882	2(1–6)	2(0–5)	0.337
Number of previous pregnancies	2(0–4)	2(0–5)	0.615	3(1–8)	2(1–5)	0.503

^a^Unpaired t-test; ^b^χ2 test; ^c^Mann-Whitney U-test. Statistically significant p-values are shown in bold.

### Impact of Sex Work on CD4^+^ T Cell Activation and Cytokines

**Figure 2:**
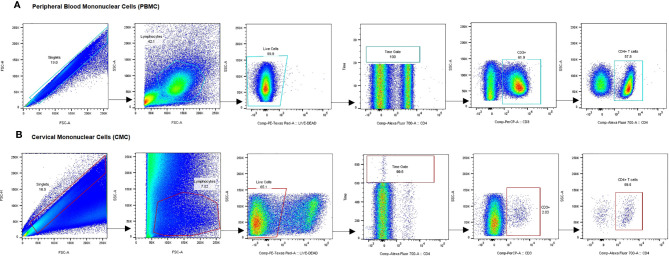
Gating strategy for analysis of CCR5 and activation marker expression on CD4^+^ T cells. Representative plots show gating on singlets, lymphocytes, live cells, internal quality control for consistency in cell flow, CD3+ cells and CD3+CD4+ cells in PBMC **(A)** and CMC **(B)**. Gating for activation markers and CCR5 was done on the CD4+ T cell fraction of PBMC and CMC. PBMC, Peripheral Blood Mononuclear Cells; CMC, Cervical Mononuclear Cells.

Using multiparameter flow cytometry, the proportion of activated CD4^+^ T cells (defined by expression of CD69, CD38, and/or HLA-DR) and CD4^+^ T cells expressing the HIV coreceptor, CCR5, was determined and compared between study groups. The gating strategy is shown in **Figure 2**. The Median Fluorescence Intensity (MFI) of these markers, which measures the intensity of marker staining on a per cell basis, was also compared. We also investigated the impact of DMPA use and sex work on the expression of proinflammatory cytokines and chemokines. As stated earlier, samples with CD3^+^ events <100 were excluded from further analysis. Based on this strict criterion, three samples were excluded: 1 FSW using DMPA and 2 non-sex workers not using any HC. The absence of these samples in the analysis did not impact the statistics since this possibility was initially factored in the power calculations.

As we had previously shown that regular sex work leads to a more quiescent immune response (20), we first started by evaluating the impact of sex work in the context of this study. To address this, sex workers using no HC were compared to non-sex workers not using HC.

### Mucosa

**Figure 3:**
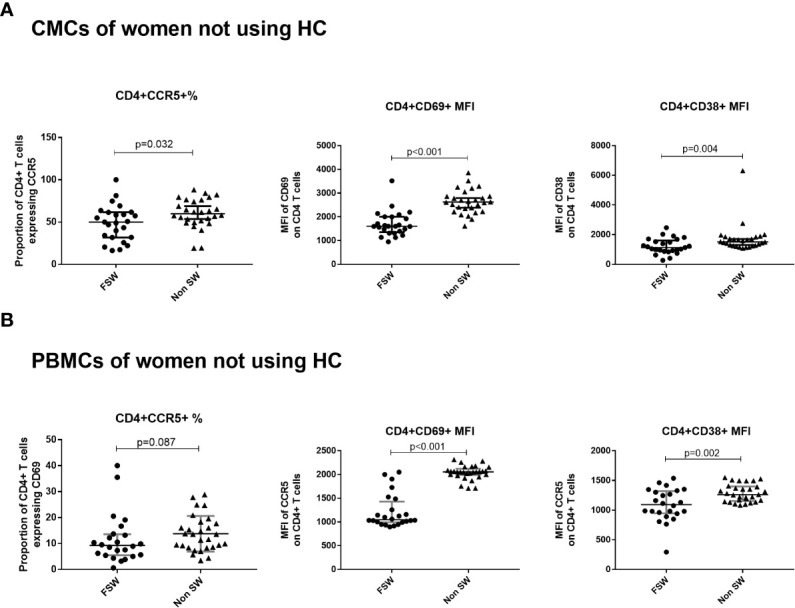
The effect of sex work on T cell activation and CCR5 expression among women not using hormonal contraceptives. **(A)** Comparison of CMCs among FSW and non-sex workers not using any HC and **(B)** Comparison of PBMCs among FSW and non-FSW using DMPA. Bars are median, interquartile range. CMC, Cervical Mononuclear Cells; PBMC, Peripheral Blood Mononuclear Cells; FSW, Female Sex Workers; MFI, median fluorescence intensity; DMPA, depot-medroxyprogesterone acetate; HC, hormonal contraception.

At the genital tract, FSWs had significantly lower CD4^+^CCR5^+^ T cells (50% vs. 59.9%, p=0.032) and MFI of CD69 and CD38 on CD4^+^ T cells (1600 vs. 2622, p<0.001; 518.1 vs. 967.5, p=0.004 respectively), (**Figure 3A**).

**Figure 4:**
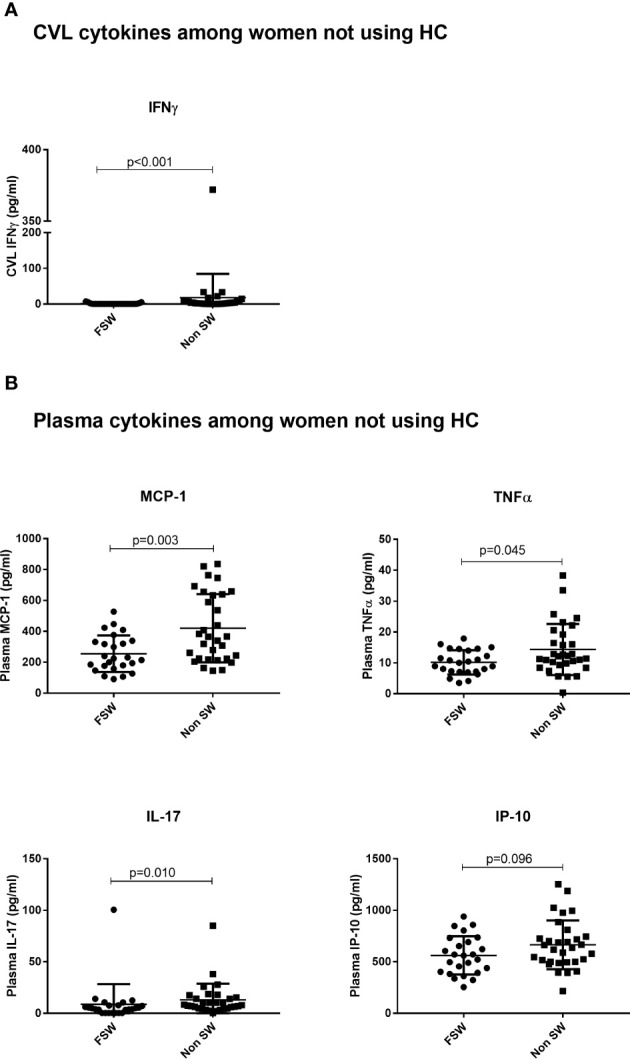
The effect of sex work on cytokine expression among women not using any hormonal contraceptives. **(A)** Genital expression of IFN*γ* between sex workers and non-sex workers not using any HC and **(B)** Comparison of plasma cytokines between sex workers and non sex workers using no HC. Bars are median, interquartile range. FSW, Female Sex Workers; DMPA, depot-medroxyprogesterone acetate; HC, hormonal contraception; CVL, Cervicovaginal Lavage.

For cytokines, IFN-*γ* was the sole cytokine with significantly lower concentration in FSWs (0.4 pg/ml vs. 3.3 pg/ml, p<0.001, **Figure 4A**) in this comparison. No differences were observed for other cytokines (**Supplementary Table 1**).

### Blood

While there were no significant differences in the proportion of activated T cells, a trend toward lower proportion of CD4^+^CCR5^+^ T cells in FSWs not using HC (9.21% vs. 13.6%, p=0.088) was observed (**Figure 3B**). In addition, significantly lower MFI of CD38 (1093 vs. 1261, p=0.002) and CD69 (1049 vs. 2056, p<0.001) on CD4^+^ T cells was observed in FSW (**Figure 3B**).

Among women not on HC, the sex worker group had lower plasma concentration of MCP-1 (224.7 pg/ml vs. 365.2 pg/ml p=0.003), TNFα (10.03 pg/ml vs. 12.13 pg/ml, p=0.045), IL-17 (4.91 pg/ml vs.7.96 pg/ml, p=0.010) and a trend toward lower IP-10 (560.4 pg/ml vs. 636.8 pg/ml, p=0.096), relative to the non-sex worker group (**Figure 4B**).

### Impact of DMPA on the Immune Response

Next, we hypothesized that use of DMPA will impact the proportions of activated T cells and HIV target cells in blood and at the FGT.

### Impact of DMPA on Mucosal Immune Response

First, we assessed the impact of DMPA use on cervical T cells and vaginal cytokines in the FGT in sex workers and non-sex workers.

#### Immunophenotyping of Cervical T Cells and Cytokine Expression Among Sex Workers

**Figure 5:**
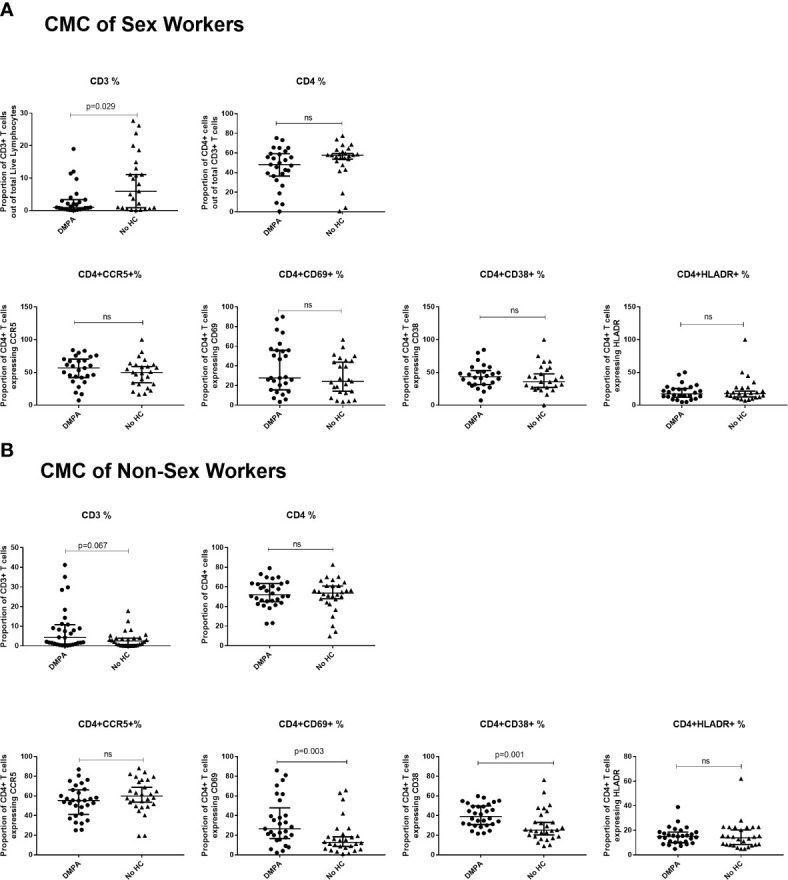
Immunophenotyping of cervical mononuclear cells (CMCs). **(A)** Comparison of CMCs of sex workers using DMPA versus no HC and **(B)** Comparison of CMCs of non-sex workers using DMPA versus no HC. Bars are median, interquartile range. FSW, Female Sex Workers; DMPA, depot-medroxyprogesterone acetate; HC, hormonal contraception; NS, not significant.

In the CMCs of FSWs, DMPA users had a significantly lower proportion of CD3^+^ T cells than FSWs not using HC (0.9% vs. 5.9%, p=0.029) but no differences were observed in CD4^+^ T cell frequency, CD4^+^ T cell activation markers and CCR5 expression (**Figure 5A**).

No significant differences in FGT cytokine expression were observed between DMPA and no HC users, except for MCP-1 which was higher in no HC users (9.9 pg/ml vs. 106.9 pg/ml, p=0.001), (**Supplementary Table 1**).

#### Immunophenotyping of Cervical T Cells and Cytokine Expression Among Non Sex Workers

In the CMCs of non-sex workers, DMPA users trended toward having a higher proportion of CD3^+^ cells (4.21% vs. 2.47%, p=0.067), and had higher proportions of CD4^+^CD69^+^ (26.5% vs. 12.6%, p=0.003) and CD4^+^CD38^+^ (39.2% vs. 25.2%, p=0.001) cells, **Figure 5B**. No differences were observed in CD4^+^ T cell frequency or other markers of activation.

Among the non sex worker women, only IFNγ (0.4 pg/ml vs. 3.3 pg/ml, p=0.001) and MCP-1 (23.7 pg/ml vs.74.8 pg/ml, p=0.011) were significantly higher in the no HC users. No significant differences in expression of other FGT cytokines were observed between DMPA and no HC users (**Supplementary Table 1**).

### Impact of DMPA on Immune Response in the Blood

Next, we determined the effect of DMPA use in the blood. A similar pattern as the one observed in the endocervical compartment was observed in the peripheral blood compartment.

#### Immunophenotyping of PMBCs and Cytokine Expression of Sex Workers

**Figure 6:**
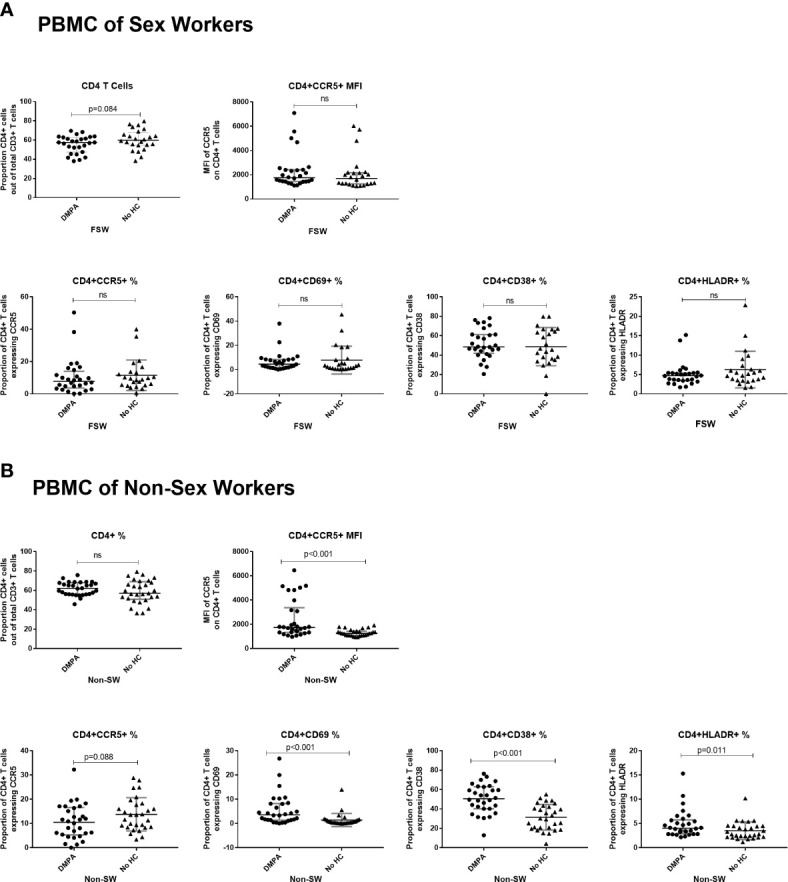
Immunophenotyping of peripheral blood mononuclear cells (PBMCs). **(A)** Comparison of CD4+ T cells of sex workers using DMPA versus no HC and **(B)** Comparison of CD4+ T cells of non-sex workers using DMPA versus no HC. Bars are median, interquartile range. FSW, Female Sex Workers; MFI, median fluorescence intensity; DMPA, depot-medroxyprogesterone acetate; HC, hormonal contraception; NS, not significant.

When comparing the immunophenotyping of PMBCs among FSW groups, there were no significant differences in expression of activation markers or the expression of CCR5 on blood-derived T cells between DMPA and No HC users (**Figure 6A**).

**Figure 7:**
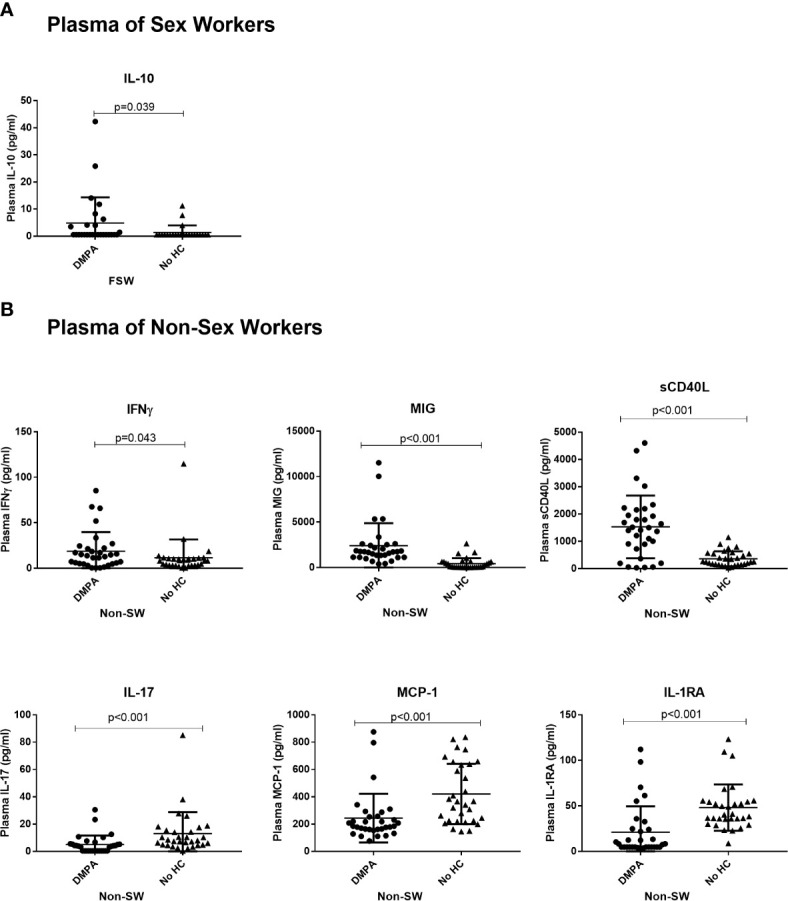
The impact of DMPA on plasma cytokine expression. **(A)** Plasma IL-10 Expression in sex workers using DMPA versus no HC and **(B)** Levels of plasma cytokines in non-sex workers using DMPA versus no HC. Bars are median, interquartile range. FSW, Female Sex Workers; DMPA, depot-medroxyprogesterone acetate.

For plasma cytokines, IL-10 (Mean 4.8 pg/ml vs. 1.4 pg/ml, p=0.039) was the only analyte increased in DMPA users compared to those not using any HC (**Figure 7A**). No differences were seen between the two FSW groups for all other cytokines (**Supplementary Table 2**).

#### Immunophenotyping of PBMCs and Cytokine Expression of Non Sex Workers

Among the non-sex worker groups, DMPA users had significantly higher expression of CCR5 on a per cell basis (MFI: 1738 vs. 1256, p<0.001). They also had higher proportions of activated CD4^+^ T cells (CD4^+^CD69^+^ T cells: 3.6 vs. 0.5, p<0.001; CD4^+^CD38^+^ T cells: 50.5% vs 30.3%, p<0.001 and CD4^+^HLADR^+^ T cells (4.0% vs. 2.8%, p=0.01), (**Figure 6B**).

Analysis of plasma cytokines among the non-sex worker group showed that DMPA use was characterized by significantly higher concentration of IFN*γ* (13.3 pg/ml vs. 8.2 pg/ml, p=0.043), MIG (1733 pg/ml vs. 118.3 pg/ml, p<0.001) and sCD40L (1457 pg/ml vs. 257.3 pg/ml, p<0.001) in the blood. On the other hand, the non-sex working women not using HC had higher levels of IL-17 (3.85 pg/ml vs. 7.96 pg/ml p<0.001), MCP-1 (184.5 pg/ml vs. 365.2 pg/ml p<0.001) and IL-1RA (7.8 pg/ml vs. 40.3 pg/ml p<0.001) than DMPA users (**Figure 7B**).

### Impact of Sex Work on the Immune Response in the Context of DMPA Use

We analyzed the combined effect of sex work and DMPA use on T cell activation and cytokine expression. To this end, FSWs using DMPA were compared to their DMPA-using non-sex worker counterparts.

### Mucosa

**Figure 8:**
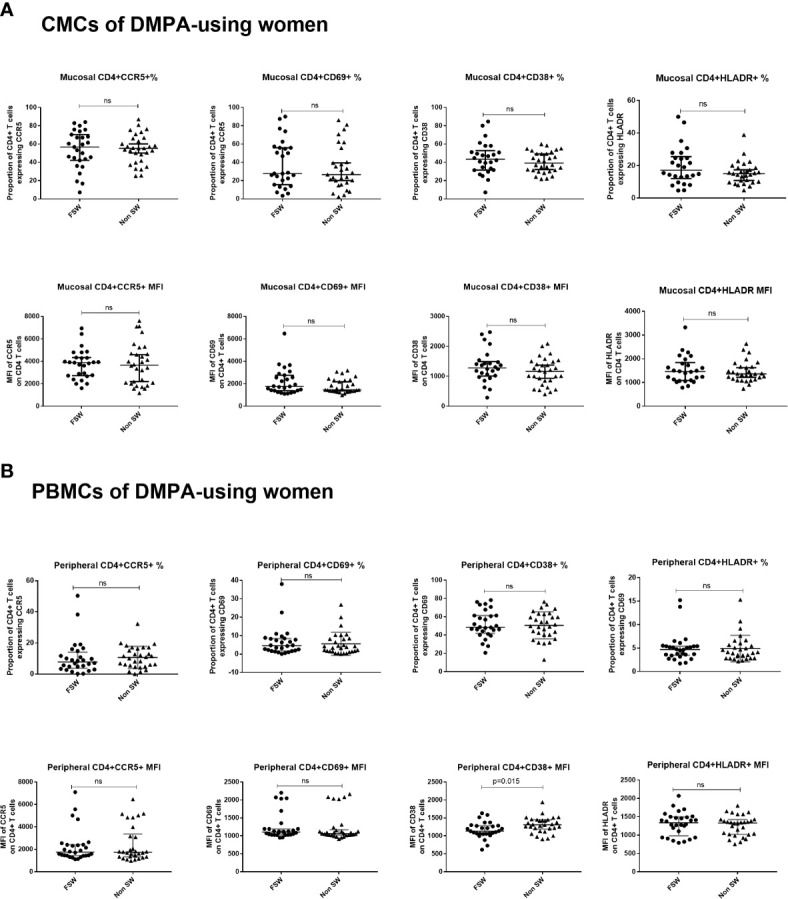
T cell activation and CCR5 expression in women using DMPA. **(A)** Immunophenotyping of CMCs sex workers and non-sex workers using DMPA and **(B)** Immunophenotyping of PBMCs sex workers and non-sex workers using DMPA. Bars are median, interquartile range. FSW, Female Sex Workers; MFI, median fluorescence intensity; DMPA, depot-medroxyprogesterone acetate; HC, hormonal contraception. NS, not significant.

This analysis revealed that FSWs and non-sex workers using DMPA had similar levels of endocervical T cell activation (**Figure 8A**), although FSWs had a diminished proportion of CD3^+^ cells (0.9% vs. 4.2%, p=0.037, **Supplementary Table 3**).

Further, no differences in mucosal cytokine expression were observed in FSWs using DMPA versus non-sex workers on DMPA except for IL-1β (**Supplementary Table 1**).

### Blood

Results of the analysis of blood-derived CD4^+^ T cells closely mirrored the observations made at the genital tract. FSWs on DMPA had activation levels similar to non-sex workers on DMPA except for level of per cell expression of CD38 where sex workers had lower levels (MFI 1133 vs. 1310, p=0.015), (**Figure 8B**, **Supplementary Table 4**).

**Figure 9:**
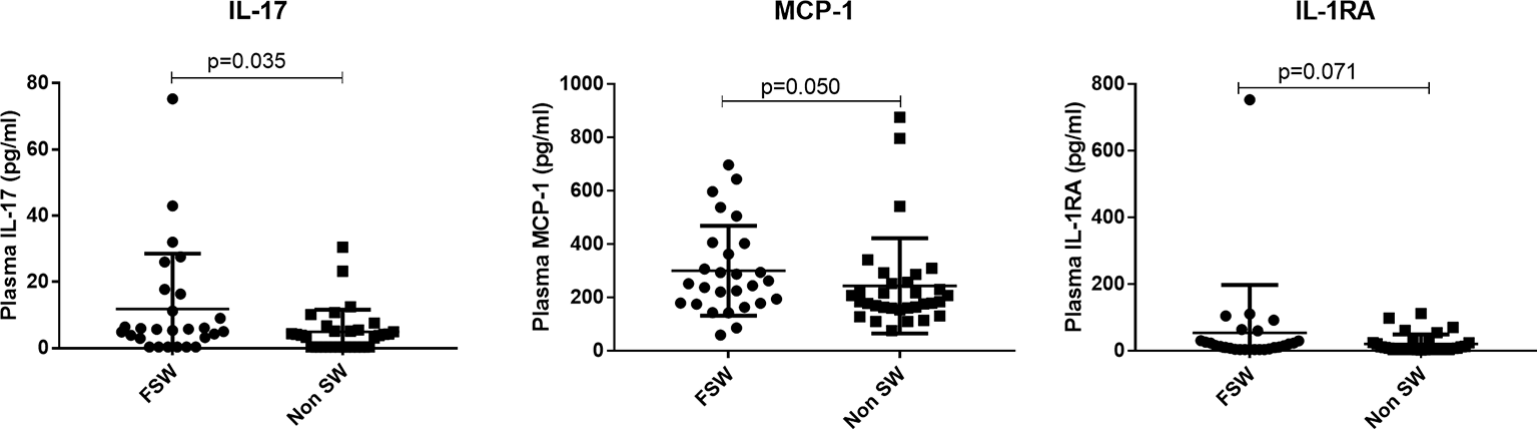
Comparison of plasma cytokine expression among women using DMPA. Plasma concentration of MCP-1, TNFa and IL-17 between sex workers and non-sex workers not using any HC. Bars are median, interquartile range. FSW, Female Sex Workers; DMPA, depot-medroxyprogesterone acetate.

For plasma cytokines, FSWs had higher levels of IL-17 (5.67 pg/ml vs. 3.85 pg/ml, p=0.035) and MCP-1 (251.8 pg/ml vs. 184.5 pg/ml, p=0.050), but lower levels of MIP-3 (10.8 pg/ml vs. 15.3 pg/ml, p=0.008). The levels of IL-1RA also trended toward being higher (15.78 pg/ml vs.7.825 pg/ml, p=0.071) in FSWs (**Figure 9**). All other plasma cytokines were similarly expressed in FSWs and non-sex workers using DMPA (**Supplementary Table 2**).

## Discussion

Understanding the biological mechanisms that mediate the relationship between DMPA use and HIV acquisition is of public health importance. This cross-sectional study was designed to investigate the role of DMPA use on inflammation and HIV target cell frequency in the blood and mucosal immune compartments of women from Nairobi, Kenya. In this study, we showed that the impact of DMPA in high-risk women such as FSWs counteracts the immune downregulation response associated with sex work, which may contribute to higher risk of HIV acquisition in a population already at high risk of HIV infection.

The first thing we noted in our study was that the majority of non-sex workers not using hormonal contraception had more years of education than the ones using DMPA. Women’s contraceptive choices are greatly shaped by their social contexts, with education being one of the major determinants (24). In Sub-Saharan countries education is strongly correlated with contraceptive uptake (24–27). This could be due to greater sexual health awareness in more educated women. However, Larsson et al. report that compared to other SSA countries, in Kenya, education matters more in women’s decision between use and non-use, rather than choice of a particular contraceptive (27). Marital status is also a factor that influences contraceptive use. In this study, less than 25% of the non-sex workers using no HC were married, while majority (70%) of the ones on DMPA were married. This finding is consistent with previous studies in Kenya that showed married women are more likely to adopt long term reversible contraception (28, 29). Since the present study was not designed to explore the contribution of education and marital status on contraceptive choices, we are limited in the interpretation of this data.

The primary aim of this study was to investigate if DMPA impacts the immune response. Among women from a community-matched general Kenyan population (called non-sex workers in this study), we observed that DMPA users had significantly increased numbers of blood-derived CD4^+^CD38^+^ and CD4^+^HLADR^+^ T cells along with increased intensity of CCR5 on CD4^+^ T cells. CD38 is a marker of mid-stage cell activation while HLA-DR appears late in the T cell activation cascade (30). This finding suggests that DMPA may drive chronic activation in peripheral circulation. It is interesting that the per cell expression of CCR5 on CD4^+^ T cells but not the relative proportion of CD4^+^CCR5^+^ was increased in the DMPA group. Apart from being the HIV coreceptor, CCR5 is a chemokine receptor that plays a critical role in the migration of effector and memory T cells to extravascular sites (31). Increased HIV coreceptor expression in response to progesterone-based contraceptives has been suggested in other studies. MPA (a longer lasting injectable form of the progestin) inhibited downregulation of CCR5 and CXCR4 on activated T cells in an *in vitro* PBMC culture (32). This surface retention of CCR5 resulted in increased HIV replication in T cells (32). Separately, Ray et al. have shown that MPA increases mRNA expression of CCR5 in endocervical tissue *via* the glucocorticoid receptor (33). Taken together, these results suggest that chronic T cell activation and a greater density of CCR5 on CD4^+^ cells in the blood may be a mechanism by which DMPA mediates T cell migration and susceptibility to HIV.

Also, in non-sex workers, proportions of endocervical CD3^+^ cells, and activated CD4^+^CD69^+^ and CD4^+^38^+^ T cells were significantly higher in DMPA users. Similar to this study, significant increase in the numbers of CD3^+^ lymphocytes in vaginal tissue following short term DMPA use has previously been reported (11) suggesting an influx of T cells to the mucosa. CD69 is one of the earliest activation markers (34), in addition to being a tissue retention marker (35, 36). Genital CD4^+^CD69^+^ T cells are preferential targets of HIV infection (37). Our observations suggest that not only does DMPA use drive activation of T cells at the FGT, it also leads to retention of a pool of activated cells in the mucosa where they can potentially be infected by HIV upon exposure.

DMPA also had an impact on the expression of innate cytokines and chemokines. Among the non-sex workers groups, the DMPA users had higher systemic expression of IFNγ, IL-10, MIG and sCD40L. This indicates that DMPA users display a higher level of inflammation compared to non DMPA users. Interestingly, women who did not take hormonal contraception showed a different profile of cytokine expression with higher level of IL-17 and IL-1RA observed. IL-1RA is an anti-inflammatory cytokine that inhibits the inflammatory activities of other IL-1 cytokines. We did not observe differences in the FGT cytokine profiles between the two groups of non sex workers. Taken together, these data indicate that in non-sex workers, DMPA drives inflammation in the blood and immune activation at the FGT, suggesting that this could increase the risk of HIV infection for women taking DMPA for contraception.

Unprotected heterosexual coitus leads to exposure of the FGT to immune stimulants such as spermatozoa, leukocytes and other signaling molecules present in seminal plasma. The FGT may also be exposed to pathogens such as HIV in the case of an infected male partner. Sexual activity thus causes an inflammatory response characterized by expression of pro-inflammatory cytokines and recruitment of activated T cells to the genital mucosa (38). Since HIV preferentially infects and replicates in activated CD4^+^ T cells, this post-coital inflammatory response creates an environment that is conducive for HIV transmission (39). It is not yet clear what the immunostimulatory capacity of seminal plasma would be in the case of multiple partners. However, sex work results in constant exposure of the FGT to immune stimulants and possibly leads an immune tolerance phenotype.

The initial years of a woman’s transition into sex work present a high risk of HIV seroconversion. In the Pumwani cohort, HIV incidence in FSW with <2 years in sex work was 3.5% compared to 1.5% for those with >2years (40). This prospective study by McKinnon et al. also found a 23% decline in risk of HIV seroconversion for each year of sex work (40). While the underlying mechanisms behind this decline in HIV susceptibility for FSWs is yet to be fully elucidated, biological factors are thought to play a role. Previous work from our group suggests that active sex work is associated with a reduction in the secretion of proinflammatory cytokines and reduced activation of T cells at the FGT (20); these observations were more marked with increasing years of sex work. Therefore, sex work and hormonal contraception are possibly intersecting immune stimulants for FSWs that directly influence susceptibility to HIV. We therefore sought to determine the effect of sex work and DMPA on the immune response by comparing sex workers versus non-sex workers not using HC, and sex workers versus non-sex workers on DMPA.

We found that FSWs using no HC have lower T cell activation levels and CCR5 expression compared to women from the general population not using HC. In the systemic compartment, FSWs had significantly lower proportion of CD4^+^CCR5^+^ cells and expression of CD69^+^ and CD38^+^ on CD4^+^ T cells (**Figure 3B**), while at the genital tract, lower proportion of CD4^+^CCR5^+^ cells and expression of the activation markers CD38 and CD69 was observed (**Figure 3A**). They also had a lower level of inflammatory cytokines and chemokines. In this study, we found lower systemic concentrations of proinflammatory cytokines (IL-17, TNFα) and of chemotactic proteins (MCP-1) in the plasma as well as lower level of IFNγ in CVL. These findings are in tandem with previous reports of low immune activation and inflammation in FSWs (23, 30). As mentioned previously, our group showed that within a year of sex work the immune response is modified toward a lower inflammatory state and the changes are more pronounced with cumulative years of sex work (20). The results of the current study are again indicating that sex work is associated with reduction in immune activation. What is leading to this immunological tolerance-like condition is not clear. One possibility is a tolerance to allo-antigen from sexual partners. Allogeneic immunity and tolerogenic responses to partner’s cells have been reported (41), suggesting that prolonged exposure to sex-related antigens from multiple sources (as in the context of sex work) may lead to a reduction of basal immune activation.

Because of this unique opportunity of enrolling sex workers and non-sex workers in the same study, it was important to understand if DMPA alters the immune response in a similar pattern between low risk and high-risk women, knowing that sex work has been shown to be such a puissant immunomodulator. Interestingly, in contrast with what was observed in FSWs not using HC, when we compared the immune response of FSWs and non sex workers using DMPA, we observed that the differences had disappeared. Indeed, they had similar levels of immune activation and inflammation. Therefore, while involvement in sex work would normally result in decreased immune activation, DMPA use leads to an increase in T cell activation and inflammation in FSWs up to the same level as the one observed in non sex workers using DMPA. This finding points to evidence that DMPA use by sex workers eliminates the strong immunomodulatory impact of sex work and could have important implications for HIV susceptibility in this group.

Indeed, as FSWs are at higher risk of HIV acquisition, any factor that contributes to a modification of the immune response toward a more activated one would have a major impact on HIV susceptibility. A number of observational studies have associated use of DMPA with HIV acquisition in African FSW (40, 42, 43). For instance, FSW in the Mombasa cohort who used DMPA in Kenya were twice as likely to acquire HIV compared to their counterparts not using any HC (43). While the mechanisms for this association may not be clear, our present study shows that the loss of immune downregulation during sex work for FSW using DMPA may underpin this observation. It is plausible that inflammation and the resultant activation of HIV target cells occurs to a greater extent in FSW using DMPA thereby increasing their risk of infection. On the other hand, those not using any HC are able to downregulate inflammation and thus have a reduced risk of HIV infection.

In a previous study from our group, we reported lower frequencies of activation markers and CCR5 on peripheral CD4^+^ T cells in sex workers using DMPA compared to those not using HC (12). In the same study, FSW using DMPA had higher levels of activation and proportions of CD4^+^CCR5^+^ T cells in cervical tissues in contrast to our findings herein. In that study, women not using HC were enrolled during the progesterone-high luteal phase of the menstrual cycle. This allowed comparison of the effect of endogenous progesterone versus exogenous progestin (DMPA). In the present study, women not using HC were recruited during the estrogen-high follicular phase. Hormonal fluctuation during the menstrual cycle is an important modulator of immune cell activation and CCR5 expression. Indeed, differential expression of chemokine receptors such as CCR5 between the follicular and luteal phases has been reported (44–46). Therefore, the apparent discordance in our findings could be explained by the varied timing of sampling of women not using HC.

This study has several strengths that should be highlighted. We sought to minimize potential confounders by excluding women with STIs and bacterial vaginosis. As exposure to seminal fluid has been associated with inflammation and recruitment of immune cells at the FGT (38), we also controlled for the presence of semen. None of the women in this study had presence of PSA in their cervico vaginal lavage at the time of sampling. We also recruited women with at least 6 months of contraceptive use for a stricter inclusion criterion. By including FSWs, we ensured that we were assessing the impact of DMPA in women who are actually at high risk of HIV infection in a country with high HIV prevalence. Further, collection of genital and systemic specimens allowed us to analyze immune markers in both the mucosal and peripheral compartments thus giving a broader picture of the effect of DMPA on the immune system.

One important limitation of this study is the time of recruitment for women who were not on any hormonal contraception. We recruited the women not using HC on days 5-10 of the menstrual cycle which represents the proliferative phase. This was done to minimize variability of the cycle among women with varying lengths of the phases of the menstrual cycle. It also easier to stage the proliferative phase as women could remember the first and last day of their menses. However, the proliferative phase is the estrogen high phase of the menstrual cycle, and the findings in these women would, therefore, not represent the dominant effect of endogenous progesterone that would be characteristic of the luteal phase in the latter half of the menstrual cycle, which has variable length and therefore more difficult to minimize variability in sampling. This limits the interpretation of the data in terms of the comparison between exogenous progestin-based HC and endogenous progesterone dominant phase of the menstrual cycle.

We also acknowledge that in this cross-sectional study, we are only able to make snapshot comparisons of women who are already using contraceptive options of their choice. We are therefore limited in terms of long term follow up that would include evaluation of the immune response pre- and post-DMPA initiation as in the case of a longitudinal study. Another potential limitation is the use of CVL for the cytokine/chemokine assays. Compared to swabs, CVL does not provide site-specific measurement and is diluted. However, lavages provide a useful sample for evaluating the overall state of inflammation at the FGT and have been collected in previous studies that enrolled women from the Pumwani Community (23, 47). Therefore, to ensure consistency with previous data and to obtain a broader picture of genital inflammation we collected CVL for evaluation of proinflammatory cytokines in this study.

In conclusion, this study has demonstrated that use of DMPA increased genital and peripheral immune activation and CCR5 expression in non-sex workers. We also show once again, that sex work decreases the level of immune activation at the genital tract. More importantly, while involvement in sex work is known to modulates the immune system, herein we show for the first time that DMPA is a more powerful immune-modulator as its use counteracts the effect of sex work on the mucosal and peripheral immune response. Taken together, these results demonstrate that DMPA alters the immune response and provide a biological mechanism underlying increased susceptibility to HIV in women, especially sex workers, using DMPA.

## Data Availability Statement

The raw data supporting the conclusions of this article will be made available by the authors, without undue reservation.

## Ethics Statement

The studies involving human participants were reviewed and approved by University of Nairobi and University of Manitoba ethics review committees. The patients/participants provided their written informed consent to participate in this study.

## Author Contributions

All authors participated in interpretation of data and critical review of the manuscript. KO was the co-study coordinator, performed technical and statistical analyses, and wrote the article. JL participated in the study design, was the co-study coordinator, and wrote the article. JO provided overall supervision of the study. DM coordinated the laboratory activities in Nairobi, Kenya. JW was involved in the initial study design of the main study. JK managed the clinical cohort. CK and KRF were the principal investigators of the study and wrote the article. CK and KRF were responsible for obtaining funding for the study. All authors contributed to the article and approved the submitted version.

## Funding

This study was funded by a team grant on mucosal immunology from Canadian Institutes of Health research (CIHR, [FRN#138657]) held by CK and KRF). KO held a fellowship from the Queen Elizabeth II Scholars Program and the CIHR International Infectious Diseases and Global Health Training Program. JW was supported by a CIHR Fellowship Award and an Ontario Women’s Health Scholars Award funded by the Ontario Ministry of Health and Long-Term Care.

## Conflict of Interest

The authors declare that the research was conducted in the absence of any commercial or financial relationships that could be construed as a potential conflict of interest.
