# Supplementary material for: Differential Elevation of Inflammation and CD4+ T Cell Activation in Kenyan Female Sex Workers and Non-Sex Workers Using Depot-Medroxyprogesterone Acetate
Source: Front Immunol. 2021 Feb 23;11:598307. doi: 10.3389/fimmu.2020.598307 (PMC7949914; doi:10.3389/fimmu.2020.598307)
Supplement: Supplementary file 1 [file Table_1.docx]

|  | **Female Sex Workers** | |  | **Non-Sex Workers** | |  | **FSW on No HC vs. non-SW on No HC** | **FSW on DMPA vs. non-SW on DMPA** |
| --- | --- | --- | --- | --- | --- | --- | --- | --- |
| **Cytokine** | **DMPA** | **No HC** | ***p-value*** | **DMPA** | **No HC** | ***p-value*** | ***p-value*** | ***p-value*** |
| IFNγ | 0.4 (0.4 - 3.63) | 0.4 (0.4 - 0.65) | 0.146 | 0.4 (0.4 - 0.4) | 3.3 (0.89 - 8.48) | 0.001 | <0.001 | 0.158 |
| IL-10 | 0.55 (0.55 - 0.55) | 0.55 (0.55 - 0.55) | 0.959 | 0.55 (0.55 - 0.55) | 0.48 (0.30 - 1.43) | 0.292 | 0.604 | 0.627 |
| IL-1β | 0.4 (0.4 - 4.97) | 1.5 (0.4 - 24.8) | 0.090 | 4.6 (0.4 - 18.6) | 12.1 (2.36 - 51.7) | 0.144 | 0.097 | 0.012 |
| IL-8 | 432.7 (136.1 - 1450) | 386 (128.8 - 2197) | 0.884 | 834.7 (421 - 2424) | 908 (273 - 2014) | 0.999 | 0.126 | 0.095 |
| MCP-1 | 9.9 (0.95 - 44.5) | 109.6 (18.53 - 262.3) | 0.001 | 23.7 (8.76 - 72.2) | 74.8 (34.8 - 290.1) | 0.011 | 0.960 | 0.097 |
| IL-1α | 45.9 (4.7 - 165.4) | 52.7 (14.83 - 143.8) | 0.704 | 99.1 (24.5 - 205.6) | 92.8 (32.7 - 234.8) | 0.951 | 0.264 | 0.130 |
| IP-10 | 19.0 (4.3 - 375) | 106.6 (4.3 - 314.4) | 0.270 | 109.9 (24.9 - 204.5) | 158.6 (48.9 - 538.4) | 0.116 | 0.103 | 0.157 |
| MIP-1α | 5.2 (1.45 - 9.41) | 5.7 (1.45 - 13.57) | 0.813 | 5.55 (1.45 - 12.95) | 1.93 (1.45 - 13.26) | 0.118 | 0.222 | 0.631 |
| MIP-1β | 5.9 (1.5 - 15.9) | 7.9 (1.5 - 15.85) | 0.944 | 7.78 (1.5 - 16.1) | 7.13 (1.5 - 13.8) | 0.769 | 0.963 | 0.696 |
| MIG | 290.6 (108.8 - 581.5) | 212.2 (104.4 - 934.8) | 0.996 | 298.1 (91.3 - 640.4) | 145 (63.6 - 916.3) | 0.777 | 0.706 | 0.809 |

Supplementary Table 1. **CVL cytokine expression among study groups.** Data are Median (Interquartile range) of cytokine concentrations in pg/ml. CVL, Cervicovaginal Lavage; FSW, Female Sex Workers; Non-SW, Non-Sex Workers, DMPA, depot-medroxyprogesterone acetate; HC, hormonal contraception.
