# Supplementary material for: Differential Elevation of Inflammation and CD4+ T Cell Activation in Kenyan Female Sex Workers and Non-Sex Workers Using Depot-Medroxyprogesterone Acetate
Source: Front Immunol. 2021 Feb 23;11:598307. doi: 10.3389/fimmu.2020.598307 (PMC7949914; doi:10.3389/fimmu.2020.598307)
Supplement: Supplementary file 2 [file Table_2.docx]

|  | **Female Sex Workers** | |  | | **Non-Sex Workers** | | |  | **FSW on DMPA**  **Vs.**  **Non-SW on DMPA** | **FSW on No HC**  **Vs.**  **Non-SW on No HC** |
| --- | --- | --- | --- | --- | --- | --- | --- | --- | --- | --- |
| **Cytokine** | **DMPA** | **No HC** | | ***p-value*** | | **DMPA** | **No HC** | ***p-value*** | ***p-value*** | ***p-value*** |
| IFNγ | 18.9 (6.15 - 40.9) | 14.7 (6.57 - 20.3) | | 0.234 | | 13.3 (4.8 - 21.9) | 8.19 (3.3 - 10.6) | 0.043 | 0.137 | 0.011 |
| IL-10 | 0.55 (0.55 - 4.15) | 0.55 (0.55 - 0.55) | | 0.039 | | 0.55 (0.55 - 3.62) | 0.55 (0.55 - 0.55) | 0.248 | 0.682 | 0.973 |
| sCD40L | 1310 (441.5 - 2181) | 1116 (719.2 - 2384) | | 0.971 | | 1457 (764.9 - 2103) | 257.3 (143.2 - 546) | <0.0001 | 0.897 | <0.0001 |
| IL-8 | 20 (7.54 - 54.7) | 12.8 (4.8 - 47.9) | | 0.445 | | 12.7 (6.47 - 26.2) | 11.7 (3.93 - 41.16) | 0.677 | 0.252 | 0.695 |
| MCP-1 | 251.8 (178.9 - 403.3) | 224.7 (168.1 - 335.7) | | 0.512 | | 184.5 (160.4 - 256.3) | 365.2 (223.4 - 639.2) | <0.0001 | 0.051 | 0.004 |
| TNFα | 12.2 (7.61 - 17.1) | 10 (7.14 - 14.26) | | 0.138 | | 12.2 (8.8 - 16.9) | 12.1 (9.3 - 19.2) | 0.644 | 0.871 | 0.045 |
| IP-10 | 594.4 (468 - 778.6) | 560.4 (397.7 - 712.1) | | 0.299 | | 680.6 (526.3 - 979) | 636.8 (499.4 - 746.2) | 0.258 | 0.321 | 0.096 |
| IL-17 | 5.67 (2.95 - 16.3) | 4.91 (0.35 - 7.7) | | 0.366 | | 3.85 (0.35 - 5.45) | 7.96 (4.81 - 15.44) | <0.0001 | 0.035 | 0.010 |
| IL-1RA | 15.8 (5.0 - 31.2) | 19.0 (5.83 - 40.6) | | 0.644 | | 7.8 (4.65 - 24.6) | 40.4 (35.4 - 54.6) | <0.0001 | 0.071 | 0.007 |
| MIG | 1400 (804.4 - 2076) | 1203 (684.9 - 1619) | | 0.266 | | 1733 (1137 - 2499) | 118.3 - 46.8 - 644) | <0.0001 | 0.234 | <0.0001 |
| MIP-3 | 10.8 (0.8 - 14.1) | 11.3 (0.8 - 23.8) | | 0.239 | | 15.3 (10.3 - 25.9) | 9.8 (3.2 - 28.8) | 0.233 | 0.008 | 0.923 |

Supplementary Table 2. **Plasma cytokine expression among study groups.** Data are Median (Interquartile range) of cytokine concentrations in pg/ml. FSW, Female Sex Workers; Non-SW, Non-Sex Workers, DMPA, depot-medroxyprogesterone acetate; HC, hormonal contraception
