# Supplementary material for: Differential Elevation of Inflammation and CD4+ T Cell Activation in Kenyan Female Sex Workers and Non-Sex Workers Using Depot-Medroxyprogesterone Acetate
Source: Front Immunol. 2021 Feb 23;11:598307. doi: 10.3389/fimmu.2020.598307 (PMC7949914; doi:10.3389/fimmu.2020.598307)
Supplement: Supplementary file 3 [file Table_3.docx]

|  | **Female Sex Workers** | |  | **Non-Sex Workers** | |  | **FSW on DMPA vs. Non-SW on DMPA** |
| --- | --- | --- | --- | --- | --- | --- | --- |
| **T cell Marker** | **DMPA** | **No HC** | ***p-value*** | **DMPA** | **No HC** | ***p-value*** | ***p-value*** |
| CD3+ % | 0.93 (0.4 - 3.33) | 5.91 (0.65 - 13.9) | 0.029 | 4.2 (0.98 - 10.8) | 2.5 (0.22 - 4.1) | 0.067 | 0.037 |
| CD4+ % | 48.2 (36.5 - 59.3) | 57.7 (49.8 - 62.4) | 0.100 | 51.9 (44.9 - 63.4) | 53.6 (44.9 - 61.1) | 0.587 | 0.169 |
| CD4+CCR5+ % | 56.7 (42.1 - 70.3) | 50 (31.8 - 61.2) | 0.314 | 55.3 (41.2 - 66.2) | 59.9 (50.1 - 75.8) | 0.197 | 0.883 |
| CD4+CCR5+ MFI | 3886 (2723 - 4334) | 2981 (2311 - 4268) | 0.204 | 3660 (2070 - 4828) | 3118 (2425 - 4301) | 0.283 | 0.784 |
| CD4+CD69+ % | 27.7 (15.5 - 55.9) | 24.1 (12.9 - 45.4) | 0.132 | 26.5 (16.7 - 47.9) | 12.7 (6.5 - 20.7) | 0.003 | 0.533 |
| CD4+CD69+ MFI | 1764 (1389 - 2766) | 1600 (1352 - 2004) | 0.228 | 1459 (1340 - 2299) | 2622 (2255 - 2864) | <0.0001 | 0.283 |
| CD4+CD38+ % | 43.4 (31.5 - 52.8) | 35.7 (26.4 - 55.7) | 0.538 | 39.2 (30.9 - 49.8) | 25.1 (18.7 - 38.9) | 0.001 | 0.445 |
| CD4+CD38+ MFI | 1276 (1009 - 1490) | 1130 (898 - 1606) | 0.470 | 1169 (815 - 1491) | 1513 (1255 - 1813) | 0.0005 | 0.234 |
| CD4+HLADR+ % | 17.2 (12.03 - 25.5) | 17.6 (11.9 - 26.7) | 0.936 | 15.0 (9.9 - 18.5) | 13.9 (8.2 - 22.4) | 0.554 | 0.231 |
| CD4+HLADR+ MFI | 1462 (1087 - 1843) | 1579 (1098 - 1905) | 0.565 | 1360 (1140 - 1661) | 1674 (1471 - 1942) | 0.006 | 0.811 |

Supplementary Table 3. **Cervical Mononuclear Cell Immunophenotypes among study groups.** Data are Median (Interquartile range). FSW, Female Sex Workers; Non-SW, Non-Sex Workers, DMPA, depot-medroxyprogesterone acetate; HC, hormonal contraception
