# Supplementary material for: Differential Elevation of Inflammation and CD4+ T Cell Activation in Kenyan Female Sex Workers and Non-Sex Workers Using Depot-Medroxyprogesterone Acetate
Source: Front Immunol. 2021 Feb 23;11:598307. doi: 10.3389/fimmu.2020.598307 (PMC7949914; doi:10.3389/fimmu.2020.598307)
Supplement: Supplementary file 4 [file Table_4.docx]

|  | **Female Sex Workers** | |  | **Non-Sex Workers** | |  |  | **FSW on DMPA vs. Non-SW on DMPA** |
| --- | --- | --- | --- | --- | --- | --- | --- | --- |
| **T cell Marker** | **DMPA** | **No HC** | ***p-value*** | **DMPA** | **No HC** | ***p-value*** |  | ***p-value*** |
| CD4+CCR5+ % | 7.8 (3.7 - 14.0) | 9.2 (5.5 - 13.6) | 0.369 | 10.5 (5.3 - 16.6) | 13.6 (8.5 - 18) | 0.088 |  | 0.392 |
| CD4+CCR5+ MFI | 1754 (1443 - 2416) | 1704 (1252 - 2173) | 0.253 | 1738 (1337 - 3373) | 1256 (1089 - 1431) | 0.0004 |  | 0.825 |
| CD4+CD69+ % | 4.5 (1.7 - 8.5) | 3.2 (1.1 - 8.9) | 0.435 | 3.6 (1.4 - 8.3) | 0.5 (0.25 - 1.6) | <0.0001 |  | 0.484 |
| CD4+CD69+ MFI | 1106 (1031 - 1195) | 1049 (995 - 1430) | 0.296 | 1066 (1022 - 1172) | 2056 - 1991 - 2122) | <0.0001 |  | 0.323 |
| CD4+CD38+ % | 48.5 (41.8 - 61.3) | 46.1 (35.3 - 65.9) | 0.539 | 50.5 (40.3 - 62.9) | 30.3 (20.2 - 42.8) | <0.0001 |  | 0.787 |
| CD4+CD38+ MFI | 1133 (1058 - 1283) | 1093 (945 - 1320) | 0.313 | 1310 (1188 - 1449) | 1261 (1154 - 1399) | 0.703 |  | 0.015 |
| CD4+HLADR+ % | 3.5 (4.7 - 5.4) | 4.9 (3.5 - 7.0) | 0.409 | 4.0 (2.8 - 5.7) | 2.8 (2.2 - 4.4) | 0.010 |  | 0.702 |
| CD4+HLADR+ MFI | 1335 (979 - 1497) | 1324 (965 - 1470) | 0.672 | 1330 (1018 - 1427) | 1615 (1511 - 1805) | <0.0001 |  | 0.541 |

Supplementary Table 4. **Peripheral Blood Mononuclear Cell Immunophenotypes among study groups.** Data are Median (Interquartile range). FSW, Female Sex Workers; Non-SW, Non-Sex Workers, DMPA, depot-medroxyprogesterone acetate; HC, hormonal contraception
